# Case Report: Infective Endocarditis Caused by *Aspergillus flavus* in a Hemodialysis Patient

**DOI:** 10.3389/fmed.2021.655640

**Published:** 2021-05-05

**Authors:** Tingting Dai, Qinghua Hu, Zhongshang Xie, Chunhui Li

**Affiliations:** ^1^Department of Pharmacy, Xiangya Hospital, Central South University, Changsha, China; ^2^The Department of Cardiovascular Surgery, Xiangya Hospital, Central South University, Changsha, China; ^3^Hospital Infection Control Center, Xiangya Hospital, Central South University, Changsha, China

**Keywords:** endocarditis, *Aspergillus flavus*, hemodialysis, valve replacement, catheter infection

## Abstract

Fungal endocarditis (FE) is a rare but fatal disease. The incidence of infective endocarditis (IE) in hemodialysis patients with catheters is thought to be obviously higher than that in the general population. We reported a case of IE caused by *Aspergillus flavus* (*A. flavus*) in a 36-year-old woman on hemodialysis. Because the blood cultures were persistently negative, so we used mNGS (Metagenomic next generation sequencing) for early clinical diagnosis. After treatment with voriconazole, the patient's condition improved rapidly. She continued oral voriconazole treatment 1 year after discharge and is in good condition. The diagnosis and treatment strategies of FE in hemodialysis patients were discussed.

## Introduction

Fungal endocarditis (FE) is a rare infection, accounting for <2% of Infective endocarditis (IE) (1–4), which is usually caused by *Candida* (5–7). *A. flavus* is a kind of Aspergillus. Although there were some reports of *Aspergillus* endocarditis (AE) in hemodialysis patients, the reports of IE caused by *A. flavus* are rare (8, 9). To the best of our knowledge, this is the first reported case employing mNGS to diagnose *A. flavus* caused IE in a patient with long term hemodialysis.

## Case Description

A 36-year-old woman was admitted to our hospital on March 3, 2020 for more than 1 months of recurrent fever. She was from Hengyang City, Hunan Province, China. Upon admission, she went through a physical examination and was found in poor general conditions. Weight: 46 kg; height: 165 cm, heart rate (HR): 115 beats/min; respiratory rate (RR): 19 breaths/min; blood pressure (BP): 112/70 mmHg; pulse oximetry (SPO2): 97%; temperature:36.6°C; cardiopulmonary auscultation with systolic murmur in mesocardium; blood count with leukocytosis (18,000/mm^3^) and deviation to the left; neutrophils 63%; Hemoglobin 62 g/L; moderate thrombocytopenia (platelets: 82,000/mm^3^), high C reactive protein (61 mg/L).

The patient was diagnosed with end-stage renal disease (ESRD) 6 years ago. She had no other disease history. In the past 6 years, the patient had regular hemodialysis twice a week *via* a right internal carotid artery catheter. On January 25, 2020, the patient began to have fever with the highest temperature of 39.6°C. There was no cough, sputum, rash and joint pain. The patient visited the local county people's Hospital, and the chest Computed Tomography (CT) results showed that the patient had pulmonary infection (Multiple nodules in the lower lobe of the right lung). The COVID-19 throat swabs PCR result and antibody result were both negative. Blood culture results of paired blood samples taken from bilateral arms prior to the empirical use of antibiotics were also negative. Due to the long-term use of catheter dialysis, the local hospital doctors considered that the fever might be caused by bacterial catheter infection. Therefore, the patient was given intravenous meropenem 500 mg 12 hourly and vancomycin 750 mg 24 hourly for empirical anti-infection treatment. However, the treatment effect was not good, and the patient still had fever. After 3 days of hospitalization, the doctor pulled out the patient's dialysis catheter and changed to direct puncture dialysis. The results of microbial culture on the tip of the removed catheter were also negative. After the removal of the catheter, the patient's condition was still not improved and still had fever, so she was transferred to our hospital to continue treatment. The venous blood samples of the patient was sent for PACEseq metagenomic next-generation sequencing (mNGS) (Hugobiotech, Beijing, China) on a Nextseq550 platform (Illumina), with results indicating *A. flavus* infection. So we adjusted the treatment to voriconazole 400 mg 12 hourly for 2 doses then 200 mg every 12 h (target trough concentration of 1.5–4.0 μg ml^−1^) and moxifloxacin 400 mg 24 hourly for anti-infection treatment, and the patient's fever symptoms were finally controlled.

**Figure 1:**
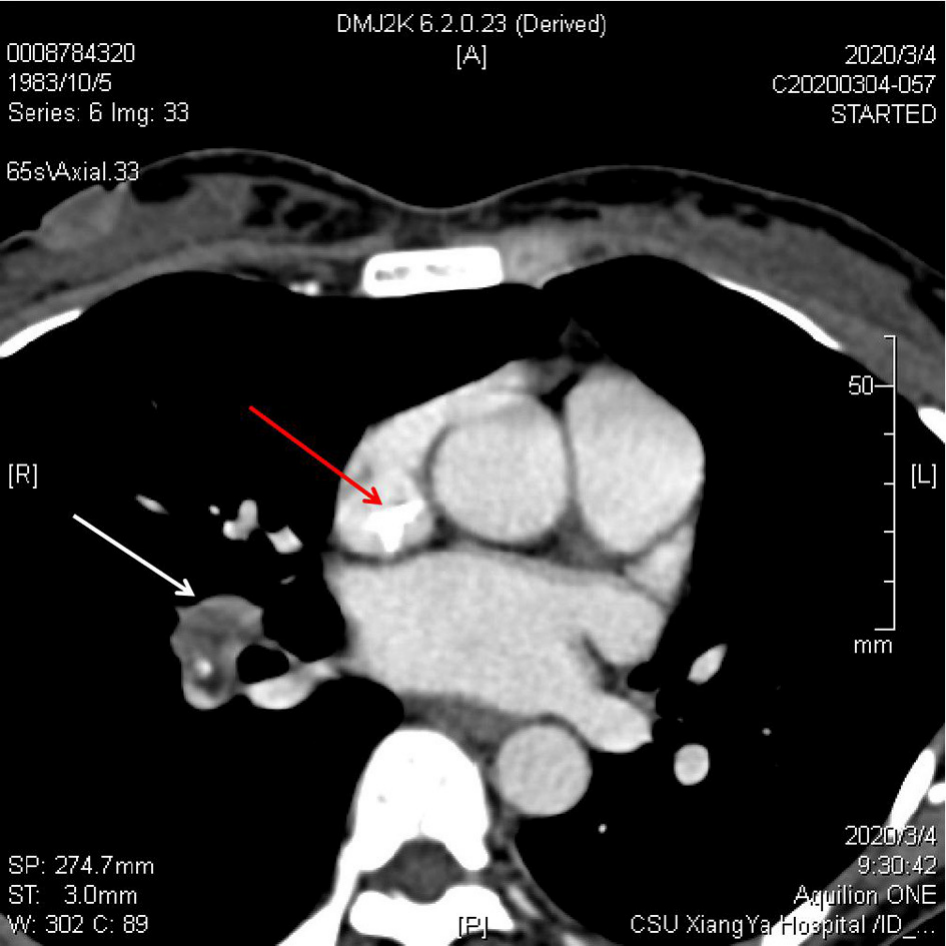
The CT findings of the patient showed calcified thrombus in superior vena cava and pulmonary artery thrombosis.

**Figure 2:**
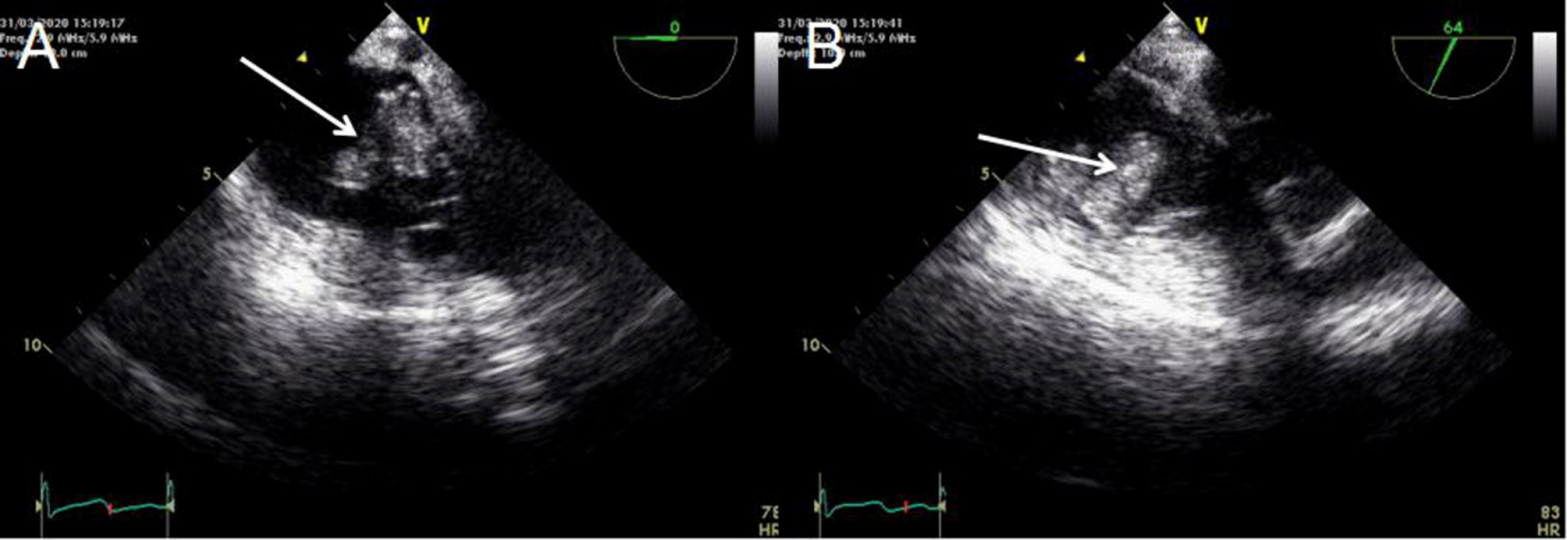
**(A,B)** Transesophageal echocardiography showing lumpy vegetations in the tricuspid valve.

The patient's Color Doppler ultrasound and superior vena cava Computed Tomography Angiography (CTA) examination showed tricuspid valve vegetation, moderate tricuspid insufficiency, scattered infection in both lungs, thrombosis in right internal jugular vein and superior vena cava, and chronic embolism of right pulmonary artery (Figure 1). Transesophageal echocardiography showed that the atrium of the anterior tricuspid valve had multiple moderate echo masses, which were about 17 × 15 mm above the apex and 18 × 10 mm near the valve root, and swayed back and forth with the opening and closing of the leaflets. Some of the masses seemed to be connected with the septal valve tip. A few masses attached to the apex of the tricuspid valve were slightly detached into the coronary sinus orifice with the leaflet when the valve was closed (Figure 2).

**Figure 3:**
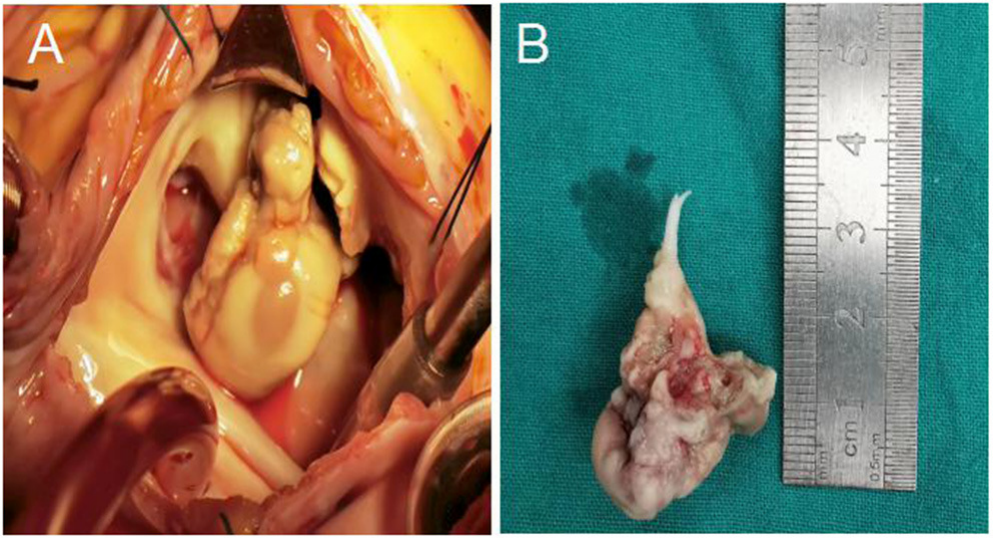
**(A)** Tricuspid valve vegetations under surgical vision; **(B)** Some tricuspid leaflets and chordae tendineae after resection.

On March 30, 2020, the patient underwent tricuspid valve removal, tricuspid valve replacement, and superior vena cava right atrium thrombectomy under general anesthesia and cardiopulmonary bypass. Histopathological examination of tricuspid valve vegetations revealed mixed bacterial thrombus in tricuspid valve with granulation tissue hyperplasia (Figure 3). Results of tissue samples sent for PACEseq mNGS confirmation (Hugobiotech, Beijing, China) and subsequent vegetative culture results from Sabouraud medium and BACT/ALERT (bioMerieux) all confirmed the presence of *A. flavus*.

**Figure 4:**
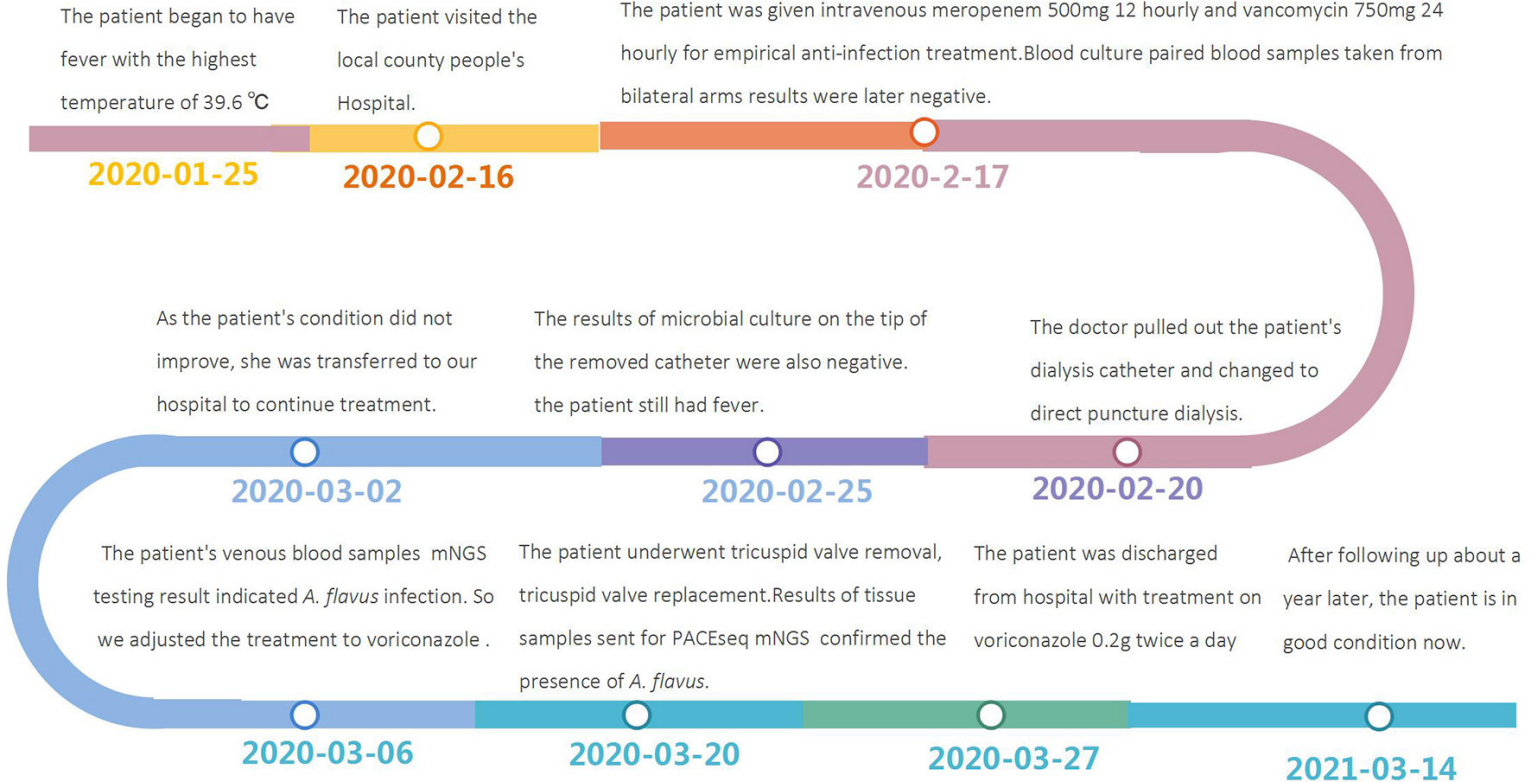
Timeline of the reported case.

Due to the improved clinical results, the patient was discharged from hospital 1 week after sugery with treatment on voriconazole 0.2 g twice a day and after following up about a year later, there were no complications or adverse events (the intuitive treatment process can be seen in Figure 4).

## Discussion

*Aspergillus* can cause fungal endocarditis, mainly in patients after organ transplantation and artificial heart valve implantation. Foreign body implantation and immunosuppression are high risk factors (2, 6). This patient with chronic renal failure, long-term dialysis treatment, combined with renal anemia, impaired immunity, and a long-term indwelling catheter in the internal jugular vein, was of a high-risk group of fungal infection. Since the common pathogens of dialysis catheter-related infections and IE are bacteria (10), empirical treatments are generally antibacterial treatments. Additionally, in *Aspergillus* infection, 93% of blood culture results were negative (11), which increased the difficulty of early diagnosis, prompt treatment and caused high mortality (2, 6). In our case, the patient's blood culture was negative at the beginning, so she was only given empirical antibacterial therapy. Fortunately, this patient has survived well after being diagnosed with *A. flavus* infection and receiving voriconazole therapy. Siddiqui BK also reported a case of IE caused by *A. flavus* in a hemodialysis patient (12). In the case they reported, because the blood cultures were persistently negative, they also used vancomycin and moxifloxacin for empirical treatment at the beginning. But unfortunately, because at that time they could not use methods like mNGS to assist in rapid diagnosis when the patient was alive, the patient developed pulseless bradycardia and expired despite resuscitation efforts. After the patient died, autopsy showed disseminated aspergillosis with a 1 × 1.5 cm vegetation on aortic valve and the grew aspergillus flavus on culture.

In cases of endocarditis with negative blood culture, for accurate and early diagnosis, 1,3-β-D-glucans (BDG) and galactomannan (GM) are promising approaches for AE to initiate antifungal therapy prior to vulvar dysfunction and heart failure. As a cell-wall biomarker of *Aspergillus*, GM has been widely used to diagnose invasive aspergillosis in neutropenic patients. In our case, because fungal infection was not considered at first, the patient was not given screening tests for fungal infections such as G tests and GM tests.

In recent years, new non-cultural methods have been developed to detect the presence of fungi in blood. These methods include mannan antigen and antibody test, polymerase chain reaction (PCR), real-time PCR target, and mNGS (13). Many studies have showed that PCR of blood may help in rapid diagnosis. Due to uncertainty and lack of standardization, recommendations of PCR for routine use in clinical practice of patients with AE remains ambiguous. In this case, we carried out the mNGS gene detection of venous blood microorganism for the patient, and the results showed that it was *A. flavus* infection. Although the results of genetic tests could not fully determine whether the patient was infected with *A. flavus*, due to the dependence of *Aspergillus* clinical diagnosis on the results of microbial culture (13), they provided strong evidence for our anti-fungal therapy. And this treatment had achieved good clinical results. The patient's tricuspid vegetative culture and mNGS results were consistent, both showing *A. flavus*, and ultimately determined that the patient was infected with *A. flavus*. mNGS is an emerging and evolving technology that started to be widely applied in clinical diagnoses. It could detect a greater number of microbial species, with high sensitivity and specificity in many studies. However, there are still difficulties, such as to identify true pathogens from environment contaminants and to distinguish viable bacteria from dead ones. More efforts are needed to further standardize and validate this method, especially with respect to sample preparation and data processing.

In our case, we didn't perform antifungal-susceptibility testing for the patient, because this testing is not a routine workup in our institution. Due to rising azole-resistant infections and differences in antifungal susceptibility profiles among strains in types and species of *Aspergillus*, performing this test is crucial for treatment (14, 15). However, since our institution does not routinely conduct this test, we had to choose the best treatment according to the guidelines. For treatment of AE, voriconazole 4 mg/kg twice daily is the first line antifungals recommended, and liposomal amphotericin B and posaconazole are the second- and third-line antifungals, respectively (16). In our case, the patient used voriconazole 400 mg 12 hourly for 2 doses then 200 mg every 12 h. After about 1 year of voriconazole treatment, the patient is still in good condition now.

There was still room to improve. In this case of suspected catheter infection, though the catheter was pulled out in time, hemodialysis was continued, with the change of position of the dialysis catheter from the right to the left internal jugular vein indwelling catheter. This might have increased the risk of re-infection. With the timely diagnosis and treatment, the patient survived and continued the hemodialysis after discharge. However, for hospitalized patients with IE in renal replacement therapy, Fernandez Cean et al. found that temporary conversion to peritoneal dialysis (PD) significantly reduced in-hospital mortality. Compared with continuous hemodialysis, the mortality rate of PD decreased from 55.5 to 8.3% (17). We will improve our treatment plan to temporarily change hemodialysis to PD during hospitalization to reduce the risk of reinfection and death, in the case of patients with reoccurring IE and requiring renal replacement therapy.

For patients with long-term indwelling catheter, we should be aware of the possibility of fungal infection. For high-risk patients with fungal infection, early identification of pathogens and antifungal treatment is very important. In our case, the patient recovered after adjusting the specific treatment according to the results of mNGS. However, after the identification of *A. flavus*, antimicrobial sensitivity examination was not conducted due to the limitation of our routine practice. In addition, continuous hemodialysis was not changed to peritoneal dialysis after the suspected catherter infection, which might increase the possibility of re-infection. Nevertheless, the antibiotics worked well fortunately, and the patient survived and continued the hemodialysis after discharge. For fungal infective endocarditis which needs surgical intervention, timely and thorough surgery combined with long-term drug treatment can achieve better results and reduce the mortality of such patients.

## Data Availability Statement

The data that support the findings of this study are openly available in NCBI (https://www.ncbi.nlm.nih.gov/) with accession number SAMN18236131.

## Ethics Statement

The studies involving human participants were reviewed and approved by the Medical Ethics Committee of Xiangya Hospital Central South University. The patients/participants provided in review their written informed consent to participate in this study. Written informed consent was obtained from the individual(s) for the publication of any potentially identifiable images or data included in this article.

## Author Contributions

TD: conception, design, and drafting the manuscript. QH: revising the manuscript. CL and ZX: conception, design, revising it critically for important intellectual content, and final approval of the version to be published. All authors read and approved the final manuscript and substantially contributed to the case report.

## Conflict of Interest

The authors declare that the research was conducted in the absence of any commercial or financial relationships that could be construed as a potential conflict of interest.
